# Adherence to infection prevention measures and vaccine uptake among pregnant women and new mothers in Sweden during the COVID-19 pandemic

**DOI:** 10.1186/s12889-026-27441-x

**Published:** 2026-04-23

**Authors:** Fanny Kelderer, Sophia Holmlund, Sven-Arne Silfverdal, Magnus Domellöf, Ingrid Mogren, Christina E. West

**Affiliations:** 1 Department of Clinical Sciences, Pediatrics Umeå University, Umeå, Sweden; 2 Department of Nursing, Umeå University, Umeå, Sweden; 3 Department of Clinical Sciences, Obstetrics and Gynecology, Umeå University, Umeå, Sweden

**Keywords:** Adherence, COVID-19, COVID-19 in pregnancy, Infection prevention, New mothers, NorthPop, Pregnant women, Sweden, Trust, Vaccine uptake

## Abstract

**Background:**

The COVID-19 pandemic was a global public health crisis that affected everyone, including pregnant women and new mothers. Previous research suggests this population adhered well to infection prevention measures, though vaccine uptake was limited due to safety concerns. Sweden’s largely voluntary and less restrictive pandemic response provided a unique context to study adherence and vaccination patterns. This study aimed to examine adherence to infection prevention measures and COVID-19 vaccination among pregnant women, new mothers, and their families.

**Methods:**

We conducted a longitudinal study using data from the prospective population-based NorthPop Birth Cohort Study in Sweden. Repeated questionnaires were distributed between June 2020 and December 2021, enabling assessment of self-reported adherence to infection prevention measures and COVID-19 vaccination uptake. Descriptive statistics were applied, and differences were analysed using Pearson’s chi-square test and independent-samples t-tests. Sensitivity analyses were performed to examine differences in adherence between participants who were pregnant during the pandemic and postpartum participants, and associations with vaccination were examined using univariable logistic regression.

**Results:**

Pregnant women and new mothers reported high adherence to infection prevention measures, peaking in December 2020 and declining thereafter for most behaviours. Compared with postpartum participants, women who were pregnant during the pandemic were less likely to limit social contacts (OR = 0.69, 95% CI: 0.49–0.97) and to stay home when experiencing cold symptoms (OR = 0.68, 95% CI: 0.49–0.93). Vaccine uptake was high (94.7%) among all participants. Participants with at least a university degree were more likely to be vaccinated than those with lower educational level (OR = 3.04, 95% CI: 1.87–4.92), and the likelihood of vaccination increased by 9% with each additional year of age (OR = 1.09, 95% CI: 1.03–1.15).

**Conclusions:**

Trust in public authorities and the healthcare system, together with a strong sense of collective responsibility, may partly explain the high adherence to recommendations and COVID-19 vaccine uptake among pregnant women and new mothers during the COVID-19 pandemic in Sweden. Sociodemographic differences in vaccine uptake highlight the need for targeted communication and support. These findings provide insights relevant for future pandemic preparedness and maternal health policy across Europe.

**Supplementary Information:**

The online version contains supplementary material available at 10.1186/s12889-026-27441-x.

## Introduction

One of the most profound public healthchallenges in recent decades was the coronavirus disease 2019 (COVID-19) pandemic [1]. Daily life was altered for all across the globe, with particularly significant consequences for individuals in vulnerable groups, including pregnant women and new parents. A mixed-studies systematic review reports that as healthcare systems faced unprecedented strain and public health measures disrupted access to maternity care, pregnancy, childbirth, and parenthood became uniquely challenging. These experiences were marked by medical uncertainty, social isolation, and increased psychological stress [2]. COVID-19 was common among pregnant women; a study conducted between March 2020 to August 2022 found that 11.1% of pregnant women in Sweden tested positive [3].

While governments and public health authorities worldwide imposed strict measures to control the spread of the severe acute respiratory syndrome coronavirus 2 (SARS-CoV-2) – including lockdowns, mask use, and mandatory vaccination – Sweden implemented a strategy largely based on voluntary individual adherence and personal responsibility [4]. In Sweden, full lockdowns were not implemented, and primary and lower secondary schools remained open. In March 2020, the National Swedish Public Health Agency issued recommendations on general hygiene measures and social distancing; however, wearing a mask was never recommended outside hospitals and other healthcare facilities. Additional recommendations included remote work, restraints on nonessential travel, a ban on gatherings exceeding 50 people, and the closure of upper secondary schools and universities, where distance learning was considered feasible [4]. These national strategies inevitably influenced the organisation of healthcare, including maternity care and child health services [5, 6]. As the knowledge of SARS-CoV-2 evolved, guidelines within healthcare were continuously updated, and these could often contribute to confusion and uncertainty among patients [7]. One example was, in the initial phase of the pandemic, that only pregnant women with medical conditions, such as severe asthma, diabetes mellitus, obesity, or hypertension, were offered COVID-19 vaccination [8]. From May 2021 onward, all pregnant women were classified as a risk group and were therefore offered COVID-19 vaccination after 12 weeks of gestation, reflecting the evolving understanding of both the medical risks of COVID-19 during pregnancy and the safety of the vaccines [8].

Outside of the healthcare system, pregnant women often adhered to precautionary measures, such as limiting social contacts, keeping distance to others, and adopting stricter hygiene practices [7, 9]. At the same time, vaccine hesitancy was relatively high among pregnant women [10], primarily due to concerns about foetal safety and the initial lack of safety data [11]. However, these studies did not investigate the issue in a context like Sweden, where adherence to infection prevention measures relied on recommendations for the public to follow, rather than legislation.

### Aims

The overall aim was to investigate adherence to national public health recommendations during the COVID-19 pandemic among pregnant women, new mothers, and their families in Sweden, focusing on reported infection prevention behaviours, and perceptions and uptake of COVID-19 vaccination.

## Methods

### Study design and setting

We conducted a study using questionnaire data from the prospective, population-based NorthPop Birth Cohort Study (NorthPop) in Sweden [12]. NorthPop is a research infrastructure at Umeå University and Region Västerbotten in the north of Sweden that consists of a database containing extensive population-based, longitudinal data from 10, 000 families. NorthPop aims to study how early life exposures, as well as parental health and lifestyle, influence future health in the child.

All eligible pregnant women in Västerbotten County were invited to participate with their partner and future child. Participants were recruited in conjunction with scheduled routine ultrasound examinations at three study sites representing urban and rural settings: Umeå, Skellefteå, and Lycksele. The inclusion started in May 2016 and was completed in September 2025. Recruitment to the cohort was ongoing during the period in which the data for this study were collected. Inclusion criteria were a viable pregnancy assessed between gestational age 14 to 24 weeks, being a pregnant woman aged 18 years or older who comprehends the Swedish language, with residence in Västerbotten county and plans to continue to reside in Västerbotten in the forthcoming years. The family is followed longitudinally until the child is at least 7 years old [12].

This study was reported in compliance with the Strengthening the Reporting of Observational Studies in Epidemiology (STROBE) guidelines for observational studies [13].

### Data collection

Data collection was conducted using web-based questionnaires. These comprised the standard NorthPop protocol (Additional file 1), and four additional questionnaires related to COVID-19, referred to as Q1 to Q4. The COVID-19 questionnaires were administered at six-month intervals between June 2020 and December 2021 and were distributed to all participating women. As a result, respondents were at different stages of the NorthPop study when completing them, including during pregnancy, early motherhood, or while their children were enrolled in daycare, which is publicly subsidized and available to all children in Sweden from age one. The participating woman reported infection prevention behaviours for both herself and her family [12]. All available responses were included in the analyses to maximize sample size.

**Fig. 1:**
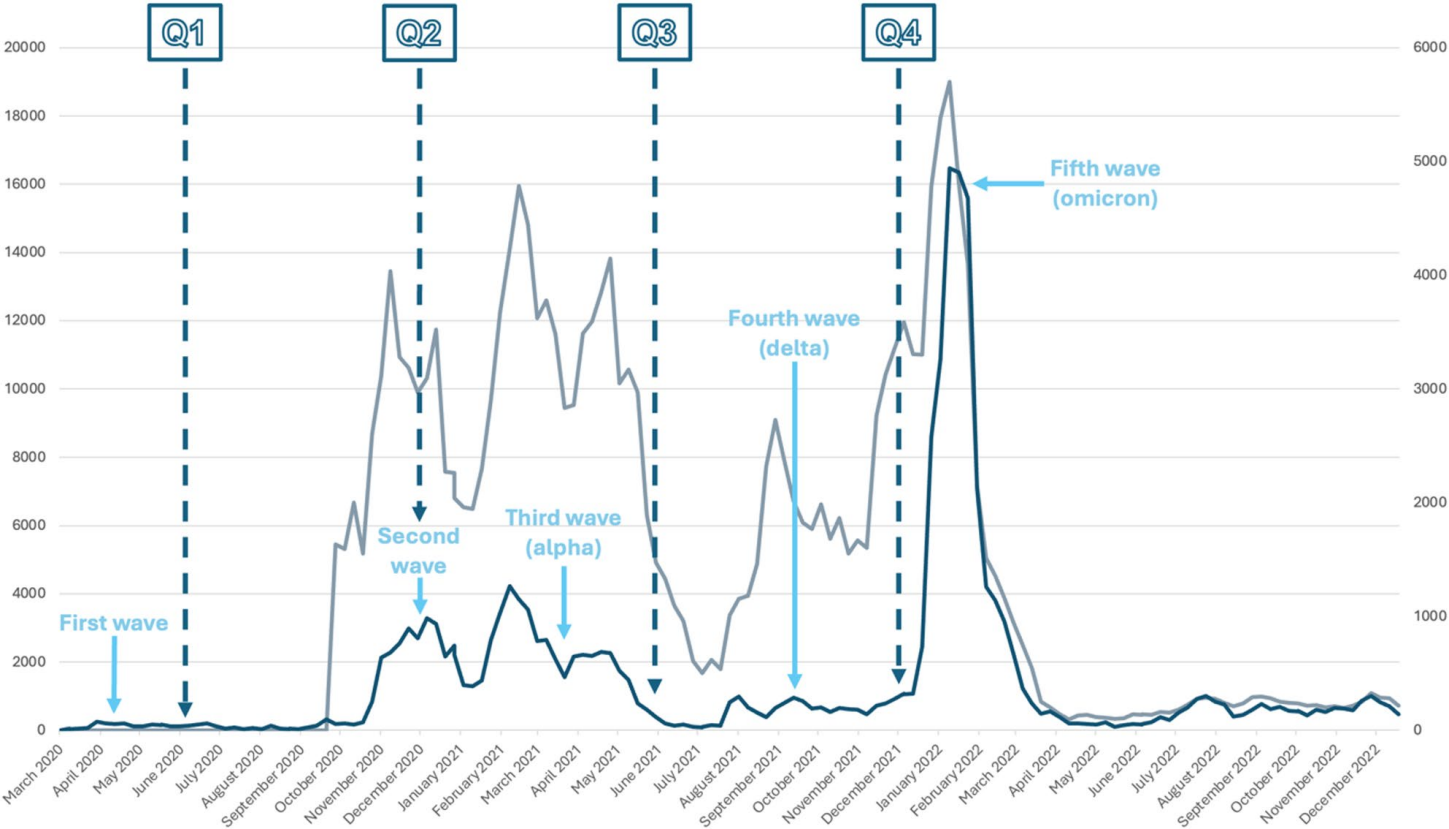
Timeline of COVID-19 questionnaires, testing, and pandemic waves in Västerbotten County, Sweden. Four questionnaires related to COVID-19, referred to as Q1 to Q4, were distributed to all participating women at six-month intervals between June 2020 and December 2021. The number of COVID-19 tests (gray, left axis) and positive tests (blue, right axis) in Västerbotten County, based on data from the Public Health Agency of Sweden. During the first wave, testing was limited to hospitalized patients, while public testing was restricted to symptomatic essential workers. Broader public testing began around late August and became available to everyone in November 2020. Vaccination against COVID-19 was offered to everyone aged 16 and older at the time of Q3 (June 2021)

Information on the woman’s age and study site was obtained from medical records in patient files. Data on maternal country of birth, socioeconomic status, household characteristics, and lifestyle factors were obtained from a questionnaire administered in early pregnancy, shortly after inclusion [12]. The COVID-19 questionnaires included items such as limiting social contacts, avoidance of public transportation, staying home when experiencing mild symptoms of a cold, working from home, hand washing and use of hand sanitizers. Questions also addressed vaccine-related topics, and how individuals perceived information from authorities, healthcare, and the media during the pandemic. See Fig. 1 for the timing of the COVID-19 questionnaires in relation to the pandemic waves.

### Definition of outcome variables

The outcome measures included a set of questions with binary responses (yes/no) capturing key infection prevention measures. Participants also reported the number of times they engaged in certain behaviours, such as handwashing frequency. In addition, some questions used ordinal response options assessing perceived intensity or degree (e.g., not noticeable, noticeable, very noticeable; and not at all, a little, somewhat, very much). Some questions allowed multiple responses, for example regarding reasons for not being vaccinated or factors motivating vaccination. Certain questions included the response option “other”, allowing participants to provide additional comments as free-text comments. These responses were also examined in the analysis.

The Public Health Agency of Sweden did not specify an exact number of daily handwashing occasions, instead, it emphasized that hands should be washed frequently and thoroughly, with soap and warm water for at least 20 s, particularly after returning home or to work, before meals or food preparation, after using the toilet, or after coughing, sneezing, or blowing one’s nose [14]. We defined an adequate handwashing frequency as seven or more times per day. This threshold is consistent with previous categorizations of daily handwashing frequency in the literature [15] (e.g., ≤ 5, 6–10, > 10) and falls within the moderate to high adherence range. Although the Public Health Agency did not provide an exact numeric recommendation, this cutoff reflects adequate handwashing frequency in line with general infection prevention guidance.

### Statistical analysis

Questions with fixed responses were analysed using descriptive statistics. Differences in categorical variables were assessed using Pearson’s Chi-square test, and differences in continuous variables were analysed using independent-samples t-tests.

Changes in participants’ reported adherence to infection prevention measures over time (Q1–Q4) were assessed using logistic regression trend analyses with linear and quadratic time terms. The linear term represents the overall trend, while the quadratic term captures non-linear changes. Odds ratios (ORs) > 1 for the linear term indicate an overall increase in adherence, whereas ORs < 1 for the quadratic term indicate a subsequent decline.

Maternal characteristics and pregnancy status were used as predictors in the regression models. Maternal characteristics included age (continuous, in years), country of birth (Sweden vs. outside Sweden), and educational level (high: university studies or higher vs. low: less than university). Pregnancy status was coded as 1 = pregnant during the pandemic and 0 = postpartum (reference group). For adherence outcomes, pregnancy status was the main predictor. For vaccination uptake, each maternal characteristic was analysed separately in univariable logistic regression models.

Sensitivity analyses were conducted to examine whether adherence to infection prevention measures differed by pregnancy status. Separate logistic regression models were constructed for each adherence outcome, with the behaviour (yes/no) as the dependent variable. To explore the relationships between the variables of interest and vaccine uptake, univariable logistic regression analyses were conducted, with each predictor analysed in a separate model and no covariates included. Results are presented as odds ratios (ORs) with their 95% confidence intervals (CI). The statistical significance level was defined as a P value < 0.05. All analyses were conducted using IBM SPSS Statistics version 30 (IBM Corp., Armonk, NY, USA).

## Results

Participating women were on average 31.0 years (SD 4.3) at the time of inclusion. More than half (54.5%) were expecting their first child (i.e., nulliparous participant at inclusion). Most women (68.7% of cases) had completed university-level education (Table 1). The number of participating women and the proportion who were pregnant increased over time due to ongoing study recruitment during the COVID-19 pandemic. Participation rates per questionnaire were 77.7% at Q1 (*n* = 2885), 71.1% at Q2 (*n* = 2959), 66.3% at Q3 (*n* = 3057), and 63.4% at Q4 (*n* = 3176). Investigating participation across all timepoints, 35.2% of women (*n* = 3972) completed all four surveys, 30.6% (*n* = 3450) completed three, 21.9% (*n* = 2475) completed two, and 12.3% (*n* = 1389) completed only one questionnaire (Table 2, placed at the end of the manuscript).

**Table 1:** Characteristics of participants

Age at inclusion (years), mean (SD)	
	31.0 (4.3)
Country of birth	
Sweden	2707 (91.0)
Other country	268 (9.0)
Place of delivery	
Umeå	2408 (83.5)
Skellefteå	405 (14.0)
Lycksele	44 (1.5)
Outside Västerbotten	28 (1.0)
Pregnant during the pandemic	
Yes	1005 (36.0)
Partner is the expected child’s biological father	
Yes	2687 (97.6)
Siblings in household	
Yes	1252 (45.5)
Type of housing	
House	1449 (51.3)
Apartment	1299 (46.0)
Farm	61 (2.2)
Other	16 (0.6)
Highest educational level^a^	
Low	84 (3.0)
Middle	798 (28.3)
High	1940 (68.7)
Employment status/current situation^b^	
On-site (commutes to a workplace)	2381 (84.4)
Remote work	127 (4.5)
Maternity leave	74 (2.6)
Sick leave	165 (5.8)
Student	267 (9.5)
Unemployed/seeking work	67 (2.4)
Other	57 (2.0)

Based on participants from Q1, with information obtained from NorthPop’s pregnancy questionnaire. Data are presented as n (%) unless otherwise indicated

^a^Low educational level was defined as elementary school or lower, middle educational level as secondary school, and high educational level as university studies

^b^Multiple answers possible

**Table 2:** Data from COVID-19 questionnaires (Q1–Q4) on participants’ self-reported SARS-CoV-2 infection and infection prevention behaviours

Participants (total)	Q1 (June 2020)	Q2 (December 2020)	Q3 (June 2021)	Q4 (December 2021)
	2885 (77.7)	2959 (71.1)	3057 (66.3)	3176 (63.4)
Pregnant during the pandemic				
Yes	1005 (36.0)	1347 (47.7)	1664 (57.1)	1041 (33.8)*
Family member infected in the last six months (strongly suspected or confirmed SARS-CoV-2 infection)				
Yes	145 (5.0)	214 (7.2)	448 (14.7)	143 (4.5)
Which family member infected				
Woman	90 (62.1)	144 (67.6)	310 (69.2)	76 (53.5)
Partner	83 (57.2)	133 (62.4)	327 (73.0)	84 (59.2)
Child	41 (32.3)	42 (24.3)	92 (24.6)	37 (28.2)
Keeping distance from others both indoors and outdoors				
Woman	2468 (88.2)	2682 (94.8)	2752 (94.2)	2561 (82.4)
Partner	2395 (86.7)	2593 (93.2)	2657 (92.6)	–
Limiting social contacts				
Woman	2344 (83.7)	2740 (96.9)	2757 (94.4)	2267 (72.9)
Partner	2217 (80.3)	2642 (94.9)	2651 (92.4)	–
Avoiding using public transportation				
Woman	2156 (77.0)	2515 (88.9)	2581 (88.3)	2263 (72.8)
Partner	2164 (78.4)	2503 (89.9)	2553 (89.0)	–
Staying home when symptoms of a cold				
Woman	2514 (89.8)	2697 (95.3)	2803 (95.9)	3039 (97.8)
Partner	2479 (89.8)	2623 (94.3)	2740 (95.5)	–
Avoiding group activities for children				
Yes	1698 (60.8)	2061 (72.9)	1991 (68.3)	1603 (51.6)
Keeping the child home from daycare when mild symptoms				
Yes	1684 (60.3)	1898 (67.2)	1977 (67.8)	2225 (71.6)
Other measure				
Woman	298 (10.6)	523 (18.5)	427 (14.6)	181 (5.8)
Partner	152 (5.5)	298 (10.7)	272 (9.5)	–
Handwashing frequency with soap and water in the last week				
0–3 times per day	158 (5.5)	178 (6.2)	267 (9.0)	272 (8.7)
4–6 times per day	573 (20.1)	677 (23.4)	781 (26.4)	865 (27.8)
7–15 times per day	1491 (52.3)	1470 (50.8)	1483 (50.2)	1487 (47.8)
> 15 times per day	630 (22.1)	567 (19.6)	426 (14.4)	489 (15.7)
Partner’s handwashing frequency with soap and water in the last week				
0–3 times per day	273 (9.7)	261 (9.2)	349 (11.9)	–
4–6 times per day	695 (24.7)	749 (26.3)	842 (28.7)	–
7–15 times per day	1343 (47.7)	1334 (46.9)	1359 (46.4)	–
> 15 times per day	467 (16.6)	454 (16.0)	326 (11.1)	–
No partner	36 (1.3)	46 (1.6)	53 (1.8)	–
Frequency of hand sanitizer use in the last week				
Never	482 (16.9)	250 (8.7)	246 (8.3)	377 (12.1)
1–6 times in the last week	1083 (38.1)	973 (33.7)	1076 (36.4)	1181 (38.0)
1–6 times per day	828 (29.1)	926 (32.1)	1008 (34.1)	1001 (32.2)
7–15 times per day	249 (8.8)	386 (13.4)	336 (11.4)	316 (10.2)
> 15 times per day	203 (7.1)	353 (12.2)	288 (9.7)	236 (7.6)
Partner’s frequency of hand sanitizer use in the last week				
Never	658 (23.7)	349 (12.5)	333 (11.6)	–
1–6 times in the last week	973 (35.1)	830 (29.7)	950 (33.1)	–
1–6 times per day	801 (28.9)	1005 (36.0)	1017 (35.4)	–
7–15 times per day	225 (8.1)	399 (14.3)	399 (13.9)	–
> 15 times per day	116 (4.2)	208 (7.5)	174 (6.1)	–
Frequency of washing the child’s hands with soap and water				
Never	163 (5.8)	184 (6.5)	162 (5.5)	157 (5.0)
1–6 times in the last week	244 (8.7)	267 (9.4)	283 (9.7)	409 (13.2)
1–6 times per day	1295 (46.2)	1392 (49.9)	1497 (51.2)	1613 (51.9)
7–15 times per day	506 (18.1)	421 (14.9)	373 (12.7)	429 (13.8)
> 15 times per day	81 (2.9)	51 (1.8)	47 (1.6)	65 (2.1)
No child < 4 years	513 (18.3)	515 (18.2)	564 (19.3)	436 (14.0)
Frequency of hand sanitizer use in the child				
Never	1512 (54.0)	1579 (55.8)	1617 (55.3)	1885 (60.6)
1–6 times in the last week	502 (17.9)	480 (17.0)	521 (17.8)	548 (17.6)
1–6 times per day	206 (7.4)	209 (7.4)	179 (6.1)	197 (6.3)
7–15 times per day	53 (1.9)	41 (1.4)	41 (1.4)	30 (1.0)
> 15 times per day	18 (0.6)	11 (0.4)	8 (0.3)	13 (0.4)
Work absence or remote work due to the pandemic				
Woman	946 (32.9)	1131 (38.4)	1370 (45.2)	–
Partner	1065 (37.0)	1288 (43.7)	1385 (45.7)	–
Reason for absence from work				
Sick leave	244 (26.0)	276 (24.9)	321 (24.0)	–
Caring for sick child	318 (33.9)	348 (31.4)	454 (33.9)	–
Quarantine rules	137 (14.6)	195 (17.6)	296 (22.1)	–
Remote work	612 (65.3)	789 (71.1)	925 (69.1)	–
Other reason	137 (14.6)	135 (12.2)	192 (14.3)	–
Reason for partner’s absence from work				
Sick leave	203 (19.4)	297 (23.8)	336 (25.0)	–
Caring for sick child	226 (21.6)	310 (24.8)	375 (27.9)	–
Quarantine rules	219 (20.9)	217 (17.4)	298 (22.2)	–
Remote work	671 (64.1)	896 (71.7)	963 (71.7)	–
Other reason	139 (13.3)	108 (8.6)	93 (6.9)	–

Data are presented as n (%)

*In the last six months

– indicates data not available

In June 2020, 5.0% of participants reported that at least one family member had had a confirmed or strongly suspected SARS-CoV-2 infection, and by June 2021 (Q3) this proportion had increased to 14.7%. In approximately two-thirds of these cases, the infected family member was a parent, while in the remaining cases it was a child (Table 2).

### Infection prevention behaviours

Overall, adherence to public health recommendations by the Swedish Public Health Agency was high throughout the pandemic (see Table 2). The proportion of participants reporting that they kept a distance from others ranged from 88.2% in Q1 to 94.8% in Q2, and was 94.2% and 82.4% in Q3 and Q4, respectively. Other infection prevention measures, including limiting social contacts, avoiding the use of public transportation, and avoiding group activities for children, followed a similar pattern. In contrast, the proportion reporting that they stayed home when experiencing symptoms of a cold – and that they kept their children home from daycare when the child had such symptoms – increased gradually from Q1-Q4 (Q1 to Q4: 89.8% to 97.8% and 60.3% to 71.6, respectively). Reported partner infection prevention measures largely corresponded with the women’s own.

### Trend analyses of reported adherence to infection prevention measures

**Fig. 2:**
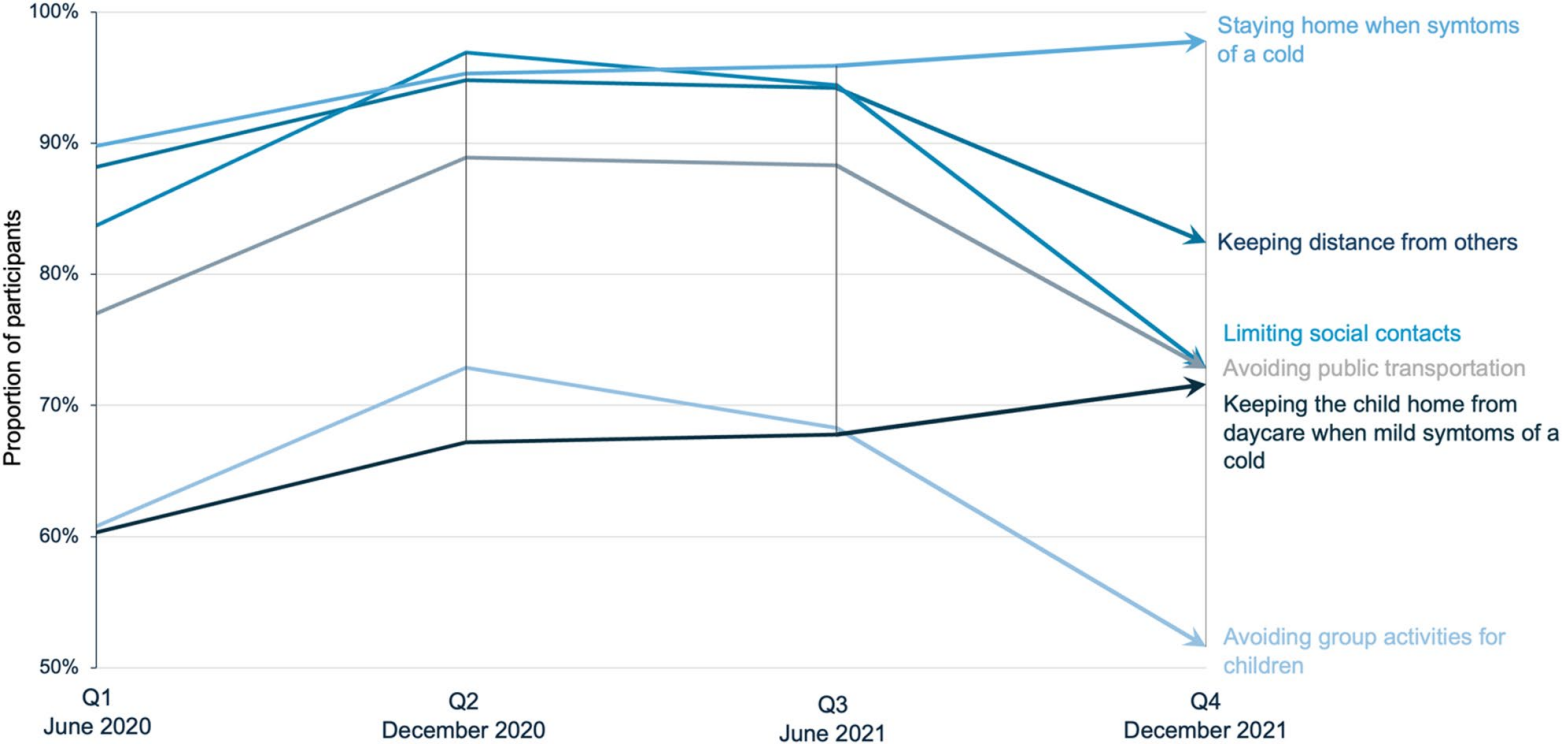
Infection prevention behaviours in pregnant women and new mothers during the COVID-19 pandemic in Sweden. Trends in infection prevention behaviour among pregnant women and new mothers in Sweden across four timepoints (Q1–Q4) during the COVID-19 pandemic, illustrating statistically significant linear and non-linear temporal patterns confirmed in regression analyses

Logistic regression trend analyses demonstrated significant temporal changes in adherence to infection prevention measures across Q1–Q4 (Table 3). The observed proportions and temporal patterns are illustrated in Fig. 2. A pronounced curvilinear pattern was observed for keeping distance (linear OR = 12.43, 95% CI 8.80–17.56; quadratic OR = 0.59, 95% CI 0.59–0.63; both *p* < 0.001), limiting social contacts (linear OR = 65.44, 45.72–93.67; quadratic OR = 0.41, 0.39–0.44; both *p* < 0.001), avoiding public transportation (linear OR = 10.12, 7.83–13.08; quadratic OR = 0.62, 0.59–0.65; both *p* < 0.001), and avoiding group activities for children (linear OR = 4.16, 3.42–5.06; quadratic OR = 0.73, 0.70–0.76; both *p* < 0.001) (Table 3).

**Table 3:** Temporal trends in SARS-CoV-2 infection prevention behaviours among pregnant women and new mothers between June 2020 and December 2021 in Sweden

OR (time) [95% CI]		OR (time^2^) [95% CI]	*P*-value (time)	*P*-value (time^2^)
Keeping distance from others both indoors and outdoors				
12.43 [8.80–17.56.80.56]		0.59 [0.55–0.63]	< 0.001	< 0.001
Limiting social contacts				
65.44 [45.72–93.67]		0.41 [0.39–0.44]	< 0.001	< 0.001
Avoiding using public transportation				
10.12 [7.83–13.08]		0.62 [0.59-0.59-65]	< 0.001	< 0.001
Staying home when symptoms of a cold				
2.48 [1.62–3.78]		0.92 [0.84–1.01]	< 0.001	0.068
Avoiding group activities for children				
4.16 [3.42–5.06]		0.73 [0.70–076]	< 0.001	< 0.001
Keeping the child home from daycare when mild symptoms				
1.35 [1.11–1.65]		0.97 [0.93–1.01]	0.003	0.138

Logistic regression trend analysis with linear (time) and quadratic (time^2^) terms. The linear term represents the overall trend in adherence, while the quadratic term captures non-linear changes over time. Odds ratios (ORs) > 1 for the linear term indicate an overall increase in adherence, whereas ORs < 1 for the quadratic term indicate a subsequent decline

In contrast, the linear trend remained significant for staying home when experiencing symptoms of a cold (OR = 2.48, 95% CI 1.62–3.78; *p* < 0.001), while the quadratic term was non-significant (OR = 0.92, 95% CI 0.84–1.01; *p* = 0.068), indicating a predominantly increasing trend. A similar pattern was observed for keeping a child home with mild symptoms (linear OR = 1.35, 1.11–1.65; *p* = 0.003; quadratic OR = 0.97, 0.93–1.01; *p* = 0.138) (Table 3).

### Adherence to infection prevention measures by pregnancy and postpartum status

We chose to conduct sensitivity analyses using data from Q2 (December 2020) because adherence to infection prevention measures was highest at this time point, and approximately half (47.7%) of participants reported being pregnant during the pandemic. This allowed for a clear assessment of whether adherence patterns differed by pregnancy/postpartum status. Compared with postpartum participants, women who were pregnant during the pandemic were less likely to limit social contacts (OR = 0.69, 95% CI: 0.49–0.97) and to stay home when experiencing symptoms of a cold (OR = 0.68, 95% CI: 0.49–0.93). No differences were observed for keeping distance from others indoors and outdoors or for avoiding public transportation (Table 4).

**Table 4:** Sensitivity analyses on adherence to infection prevention measures by pregnancy and postpartum status

Self-reported infection prevention behaviour	OR	95% CI	*p*-value
Keeping distance from others both indoors and outdoors	0.84	0.62–1.12	0.235
Limiting social contacts	0.69	0.49–0.97	0.034
Avoiding using public transportation	0.84	0.66–1.08	0.166
Staying home when symptoms of a cold	0.68	0.49–0.93	0.017

Data from Q2. Pregnancy status was defined as pregnant during the pandemic versus postpartum (reference group). Odds ratios (ORs) were estimated using logistic regression with each adherence outcome as the dependent variable. OR < 1 indicates lower odds for pregnant participants compared with postpartum participants

### Adherence to handwashing recommendations

The proportion of participants reporting an adequate handwashing frequency decreased over time, from 74.4% in Q1 to 63.5% in Q4. Corresponding figures for participants reporting washing their children’s hands to the same extent decreased from 21.0% in Q1 to 14.3% in Q3, with a slight increase to 15.9% in Q4 (Table 2). We compared participants who washed their hands seven or more times per day with those who did not. Those who reported being adherent to handwashing recommendations were slightly older (31.1 ± 4.4 versus 30.5 ± 4.1 years; p = < 0.001) than non-adherent participants. Participants living in houses or apartments reported washing their hands more frequently compared to those living on farms (*p* = 0.022). No significant differences were observed based on country of birth (Sweden or other country; *p* = 0.902), educational level (*p* = 0.484), siblings in the household (*p* = 0.131), or work location (home versus remote work; *p* = 0.835) (Table 5).

**Table 5:** Analysis of differences in characteristics between participants who reported adherence to the Public Health Agency’s handwashing recommendation and those who did not

	Compliance to handwashing recommendations^a^		
	Yes	No	
	*N* = 2121 (74.4)	*N* = 731 (25.6)	
Predictor	n (%)	n (%)	*P*-value
Age at inclusion (years), mean (SD)^b^	31.1 (4.4)	30.5 (4.1)	< 0.001
Country of birth			
Sweden	1898 (74.4)	652 (25.6)	0.902
Other country	181 (74.8)	61 (25.2)	
Siblings in household			
Yes	935 (75.7)	300 (24.3)	0.131
No	1088 (73.2)	399 (26.8)	
Type of housing			
House	1091 (76.1)	342 (23.9)	0.022
Apartment	942 (73.4)	342 (26.6)	
Farm	38 (63.3)	22 (36.7)	
Other	9 (56.3)	7 (43.8)	
Highest educational level^c^			
Low	64 (76.2)	20 (23.8)	0.484
Middle	601 (75.9)	191 (24.1)	
High	1412 (73.8)	502 (26.2)	
Remote work			
Yes	29 (69.0)	13 (31.0)	0.835
Partly	62 (74.7)	21 (25.6)	
No	1691 (74.4)	583 (25.6)	
Does not work	295 (75.4)	96 (24.6)	

Analysis based on reported data on handwashing in Q1. Data are presented as n (%) and P-value based on Pearson Chi-Square test unless otherwise indicated

^a^Defined as handwashing with soap seven times per day or more, in accordance with the Public Health Agency’s guidelines during the COVID-19 pandemic. Self-reported questionnaire data, answered during the pandemic.

^b^Independent-samples t-test

^c^Low educational level was defined as elementary school or lower, middle educational level as secondary school, and high educational level as university studies

### Other infection prevention measures

Participants described a wide range of additional precautionary behaviours in their free-text comments, referring both to their own actions and those of their partners. In Q1, 10.6% of participants selected “other” and elaborated on their behaviours. The most mentioned actions included adopting new shopping routines, such as shopping alone or during off-peak hours, ordering groceries online, and avoiding physical stores. Participants also frequently described being extra careful with hygiene, limiting travel or leaving home only when necessary, and working from home whenever possible.

Over time, the distribution of reported behaviours shifted. In Q4, 5.8% of respondents provided free-text comments, and analyses showed that the use of face masks became increasingly reported during this period, despite masks not being officially recommended by the Public Health Agency in Sweden. Measures such as strict avoidance of physical stores or any in-person social interactions were mentioned less frequently.

### Work absence or remote work due to pandemic

Before the COVID-19 pandemic, 4.5% of participants reported working remotely (Table 1). In the following COVID-19 questionnaires (Q1-Q3), the proportion of women reporting work absence or remote work due to the pandemic increased over time (from 32.9% to 45.2%), and their partners demonstrated a similar pattern (37.0 to 45.7%). The most common reason for work absence for both parents was remote work, followed by sick leave, quarantine, and temporary parental leave to care for sick children (Table 2). Other reasons, reported by 14.6% of participants in free-text comments in Q1, mainly included remote studies and parental leave. Participants also mentioned pregnancy-related reasons, short-term furlough, having a family member classified as being at risk of severe COVID-19, or being on sick leave for non-COVID-19-related reasons.

### Vaccine uptake

The fourth and final questionnaire (Q4) included vaccine-related questions. A total of 94.7% reported having received vaccination against COVID-19. Vaccination uptake was slightly lower (91.3%) among participants who had been pregnant during the last six months. For participants who were not vaccinated, the primary reasons were concerns about potential side effects for themselves (37.4%) and their foetus (39.9%) (Table 6, placed at the end of the manuscript). A total of 27.6% of participants reported “other reason” and provided additional details in free-text comments. The most reported reasons, from most to least frequent, were feeling it was unnecessary or not wanting to, breastfeeding, and having recently had the COVID-19 infection. Examples of free-text comments include: “Because I am young and healthy and will most likely manage a potential COVID-19 infection”, “I am breastfeeding and do not want my child to experience possible side effects from the vaccine”, and “Had COVID, but want to wait for an updated vaccine and for more research on side effects.”

**Table 6:** Participants’ reported COVID-19 vaccination status, motivations, trust, and information sources in December 2021

Have you been vaccinated against COVID-19?	
Total respondents	3168 (100)
Yes	3000 (94.7)
No	164 (5.2)
Not sure	4 (0.1)
If pregnant in the last six months, have you been vaccinated against COVID-19?	
Total respondents	1041 (100)
Yes	950 (91.3)
No	88 (8.5)
Not sure	3 (0.3)
If no, reasons for not getting vaccinated?^a^	
Total respondents	163 (100)
I have already had the infection and therefore do not need the vaccine	9 (5.5)
I am pregnant and concerned that the fetus may be affected by side effects	65 (39.9)
I am concerned about experiencing side effects	61 (37.4)
I have an underlying medical condition	5 (3.1)
I have not had the time	9 (5.5)
I plan to get vaccinated but have not done so yet	42 (25.8)
Other reason	45 (27.6)
What would motivate or motivated you to get the vaccine?^a^	
Total respondents	3163 (100)
To protect my health	2800 (88.5)
To protect the health of my family and friends	2919 (92.3)
To protect my colleagues’ health	2396 (75.8)
To protect public health	2675 (84.6)
To return to work or school	1369 (43.3)
To resume social activities	2057 (65.0)
To resume travel	1431 (45.2)
Encouragement from others to get vaccinated	453 (14.3)
Other reason	139 (4.4)
How much do you trust the Public Health Agency’s recommendation to get vaccinated against COVID-19?	
Total respondents	3160
Not at all	62 (2.0)
A little	231 (7.3)
Somewhat	880 (27.8)
Very much	1987 (62.9)
To what extent have the following sources of information influenced your viewpoint towards vaccination?	
Total respondents	3143 (100)
News from national television or radio	
Not noticeable	1167 (37.1)
Noticeable	1541 (49.0)
Very noticeable	435 (13.8)
Government authorities	
Not noticeable	585 (18.6)
Noticeable	1598 (50.8)
Very noticeable	960 (30.5)
Social media and internet forums	
Not noticeable	2055 (65.4)
Noticeable	892 (28.4)
Very noticeable	196 (6.2)
Discussions with friends and family	
Not noticeable	997 (31.7)
Noticeable	1633 (52.0)
Very noticeable	513 (16.3)
Healthcare professionals	
Not noticeable	1335 (42.5)
Noticeable	1341 (42.7)
Very noticeable	467 (14.9)
Other source	168 (5.3)

Data from Q4 and presented as n (%)

^a^Multiple answers possible

The primary motivations for vaccination were to protect the health of family and friends (92.3%), protect one’s own health (88.5%), contribute to public health (84.6%), and protect colleagues’ health (75.8%). Among participants, 4.4% reported “other reasons” and, in free-text comments, most mentioned wanting reassurance that the vaccine is safe and protects the foetus or child.

Trust in the Swedish Public Health Agency’s recommendations for COVID-19 vaccination was high, with over 90% of participants reporting that they trusted the guidance *somewhat* to *very much*. Other sources that influenced opinions on vaccination were national news sources and healthcare professionals, and to the same extent, also discussions with friends and family (Table 6). A total of 5.3% of participants reported “other sources,” with some elaborating in free-text comments that reading research reports or experiences from working in healthcare further informed their approach and behaviour.

### Associations between maternal characteristics and COVID-19 vaccination

Younger maternal age and lower educational level were statistically significantly associated with being unvaccinated. Participants with at least a university-level education had three times higher odds of being vaccinated compared to those with lower education (OR = 3.04, 95% CI 1.87–4.92). Maternal age was also significantly associated with an increased likelihood of vaccination, with the odds increasing by 9% per additional year of age (OR = 1.09, 95% CI 1.03–1.15). Participants who had been pregnant during the last six months had significantly lower odds of being vaccinated (OR = 0.33, 95% CI 0.24–0.47) (Table 7).

**Table 7:** Univariable logistic regression of predictors for COVID-19 vaccination by December 2021 in pregnant women and new mothers in Sweden

Predictor	OR	95% CI	*P*-value
Age (per year increase)	1.09	1.03–1.15	0.002
Born in Sweden (vs. outside Sweden)	2.18	1.38–3.43	< 0.001
High educational level^a^ (vs. low)	3.04	1.87–4.92	< 0.001
Pregnant during the last six months (vs. not pregnant)	0.33	0.24–0.47	< 0.001

^a^High educational level was defined as university studies or higher

### Participant analyses

Approximately one third of the participants who received the COVID-questionnaires did not respond. Further analysis demonstrated that participants in the non-response group were significantly younger (32.4 ± 4.9 versus 32.9 ± 4.5 years; *p* = 0.004) and had significantly lower educational level (response rates: 47.9% for low, 63.5% for medium, and 71.7% for high education; *p* < 0.001). There were also significant differences in response rates between study sites, with a lower response rate in the largest town Umeå (66.4%), compared with the smaller towns of Skellefteå (76.9%) and Lycksele (70.2%; *p* < 0.001). Families including siblings demonstrated a significantly lower response rate (68.2% versus 71.4%; *p* = 0.035). No differences were observed between participants born in Sweden and those born in other countries, or in housing type (Additional file 2).

## Discussion

Our findings indicate that pregnant women and new mothers in Sweden generally demonstrated strong adherence to infection prevention measures and reported a high uptake of COVID-19 vaccination during the early and mid-phases of the pandemic. Pregnant women demonstrated significantly lower adherence to certain infection prevention measures compared with postpartum women. These differences may reflect variations in risk perception where new mothers may feel a stronger need to protect their newborns and themselves, whereas pregnant women might perceive some measures as less feasible or less urgent, and may consider the foetus to be relatively protected in utero. These patterns emerged in the context of Sweden’s unique pandemic approach, which relied more on individual voluntary adherence, individual responsibility, and public trust than on strict lockdowns and legal mandates.

The data for this study were mainly collected prior to the emergence of the Omicron variant, and this period was characterised by evolving knowledge of SARS-CoV-2, the initial rollout of COVID-19 vaccines, and specific recommendations for pregnant women, which changed over time. In Sweden, vaccination was initially offered to selected risk groups, including pregnant women with comorbidities, and shortly after to all pregnant women from May 2021 [8]. As the pandemic progressed into later phases, changes in risk perception across the population, along with evolving public health recommendations, may limit the generalisability of our findings to subsequent stages of the pandemic.

Understanding the factors that influence adherence to infection prevention measures is critical, as adherence varies across individuals and populations. Our study population consisted primarily of young and healthy individuals who, although generally low-risk, were nevertheless classified as a risk group during pregnancy [16]. In addition to this formal classification, concerns about the health of the foetus and the newborn were prominent and likely played a significant role in shaping their behaviour. Previous studies, using both quantitative and qualitative approaches, have shown that individuals were more likely to comply with COVID-19 prevention measures if they experienced some degree of fear of the SARS-Cov-2 virus [17–19]. However, a Norwegian study found that people who believe infection control measures are effective are the most likely to comply [20]. In high-resource settings such as Sweden, cultural factors, including a high level of public trust in governmental institutions and confidence in the accessibility and quality of health services, likely support adherence to public health measures. The preparedness of the healthcare system, alongside consistent financial and political commitment to public health, may contribute to higher levels of compliance. Given the high level of trust in the National Swedish Public Health Agency and the healthcare system reported by participants, these contextual factors likely contributed to the strong adherence observed in our study.

Our study demonstrated a remarkably high willingness to receive the COVID-19 vaccine (94.7%) among participants, exceeding reports from several other countries in both high- and low-resource settings. A systematic review including over twenty-five thousand women worldwide shows that the proportions of women willing to be vaccinated during pregnancy and breastfeeding were 49.1% and 61.6%, respectively, with similar patterns observed in both low- and high-resource countries [11]. In line with this, our findings indicate that pregnant women were less likely to be vaccinated compared with women in the postpartum period. In our study, participants who remained unvaccinated were generally younger and reported a lower educational level. This is consistent with previous studies, which have also identified a low socio-economic level as a significant predictor of vaccine hesitancy or refusal [8, 21]. Participants reported hesitancy due to concerns about the safety and effectiveness of vaccines, similar to findings from a study on pregnant women in Croatia [22]. Concerns expressed by healthcare providers themselves may further influence pregnant women’s willingness to be vaccinated, and this is likely to affect postpartum women as well, even though they were not included in the review by Etti et al. [23] To reach individuals who are less likely to follow public health recommendations or to accept vaccination, information must be communicated in an accessible and understandable way. Given the results of our study, tailored strategies should focus on capturing attention, simplifying messages, and making engagement effortless, as suggested by a study in New Zealand [24].

Although adherence to infection prevention measures was generally considered high among participants, it declined over the course of the COVID-19 pandemic. This gradual decrease may reflect behavioural fatigue, consistent with a previous study showing that the individuals’ patience with recommendations diminished and their desire to return to a more normal daily life increased [25]. This concept aligns with “pandemic fatigue” as recognized by the World Health Organization (WHO), describing a demotivation to follow recommended protective behaviours during prolonged public health crises [26]. Another possible explanation is that by the time of the final questionnaire (Q4) in December 2021, COVID-19 vaccination was widespread and well established, potentially reducing participants’ perceived need for strict preventive behaviours. In contrast, earlier evidence indicates that vaccination did not lead people to relax their behaviour, as a UK study found no reduction in compliance after vaccination and even noted increases in adherence over the same period [27]. Moreover, the distribution of reported infection preventive measures changed over time. For instance, the use of face masks increased from Q1-Q4 even though their use was not officially recommended in Sweden [4]. This may have been influenced by international practices, as face masks were widely promoted in other countries, and they may have provided a sense of security or justification for engaging in activities that participants had previously avoided to prevent infection.

### Methodological considerations

An important methodological strength of this study is its prospective, population-based design, which supports the robustness of the observed associations. Approximately 60% of all pregnant women in Västerbotten County participates in NorthPop, indicating that the sample is likely to be broadly representative of the regional population and may help reduce the risk of selection bias. The participants come from both urban and rural areas, including cities of varying sizes. This diversity may contribute to a broader representation of the population, suggesting that the study setting does not differ substantially from other regions in Sweden in terms of key demographic and healthcare characteristics. However, regional differences in sociodemographic composition, healthcare access, and attitudes towards public health recommendations may still exist, and these should be considered when interpreting the generalisability of the findings.

The longitudinal design, with four questionnaires (Q1–Q4) distributed at different time points during the COVID-19 pandemic, enabled us to examine temporal trends in behaviours and adherence to infection prevention measures. The questionnaires were distributed during periods of varying pandemic intensity, capturing responses across different stages of the outbreak. Collecting data at each time point possibly reduced recall bias, as participants reported their behaviours close to when they occurred rather than retrospectively over longer periods. However, the composition of the study sample changed over time, with the proportion of pregnant participants increasing due to ongoing recruitment during the COVID-19 pandemic. Given that participants who had been pregnant during the pandemic and postpartum participants showed differences in certain infection prevention behaviours, these changes in sample composition may partly influence the observed trends in adherence over time. While our analyses suggest that main patterns are robust, temporal trends should be interpreted with consideration of the evolving cohort composition.

It is possible that adherence to infection prevention measures was over-reported due to socially desirable responding, with participants providing answers they perceived as “correct” or expected. Such bias would typically result in a modest overestimation of adherence levels. However, we believe that the observed temporal trends likely reflect changes over time, even if absolute proportions are moderately inflated. It is uncertain how socially desirable responding would manifest in a context like this study, where responses were anonymous and there was no obvious benefit in reporting adherence more positively [28].

Approximately one-third of the participants did not respond to the questionnaire, which constitutes a limitation of the study. The participant analyses show an underrepresentation of specific population groups; younger participants and those with lower level of education. Furthermore, participation in NorthPop requires the ability to read and understand Swedish, which excludes a considerable proportion of the foreign-born population. This linguistic and cultural selection likely limits the generalizability of our findings to the broader population.

In addition to these factors, selection bias could have been introduced if eligible participants with particular attitudes toward health, healthcare engagement, or vaccination were more likely to participate in the study. People who choose to take part in research are often motivated by trust in healthcare services [29], and these characteristics may correlate with higher vaccine willingness, most likely inflating the overall vaccination uptake observed in our study. Conversely, individuals with vaccine hesitancy or limited connection to healthcare systems may be less likely to participate, which could further bias the results of a study.

Taken together, these factors suggest that the study sample is likely healthier, more health-engaged, and more socioeconomically advantaged than the source population of Västerbotten, Sweden. Consequently, the results should be interpreted with caution when generalizing to underserved or socially marginalized groups. Furthermore, these findings are situated within a high-resource setting, and patterns of adherence and vaccination uptake may differ substantially in low-resource settings, where access to healthcare, public health infrastructure, and trust in authorities can be more limited.

### Ethical considerations

This study involved pregnant women and new mothers, a population considered potentially vulnerable due to their unique circumstances and the psychosocial challenges associated with pregnancy and early motherhood during the COVID-19 pandemic. All procedures adhered to the principles of the Declaration of Helsinki [30]. Participants were fully informed about the study’s purpose, procedures, and voluntary nature. Confidentiality and data protection were strictly maintained, and participants were free to withdraw at any time without consequences.

## Conclusions

Our study found that adherence to infection prevention measures and COVID-19 vaccination uptake were generally high among pregnant women and new mothers. Participants who remained unvaccinated were younger, had lower educational attainment, and more often pregnant at the time of the data collection, underscoring the need of targeted outreach to these subgroups. These findings were observed in the Swedish context, where high levels of trust in public authorities and a strong sense of collective responsibility likely supported compliance.

A recent commentary underscores that pregnant and breastfeeding women face distinct vulnerabilities during infectious disease outbreaks and must be better integrated into preparedness efforts, including through risk communication that directly addresses them in accessible formats to support vaccine uptake and counter misinformation [31]. As future pandemics are widely regarded as inevitable [32], rendering population-level experiences during the COVID-19 pandemic highly relevant. Nurturing trust and public engagement through transparent communication and education may be more effective in promoting adherence than relying on strict or mandatory measures. Accessible and affordable healthcare services play a key role in supporting adherence, as people are better positioned to follow recommendations when they can obtain care without financial barriers.

## Supplementary Information

Additional file 1. The NorthPop timeline

Additional file 2. Additional Table 1. Participation analysis in relation to maternal characteristics

## Data Availability

Original data are held by the NorthPop project team. Due to Swedish data storage laws, we cannot make the data publicly available. Pseudonymized data may be provided upon requests to the corresponding author, if providing a reasonable proposal and if an appropriate data sharing agreement with Umeå University can be established.

## Abbreviations

CI: Confidence interval
COVID-19: Coronavirus disease 2019
NorthPop: NorthPop Birth Cohort Study
OR: Odds ratio
SARS-CoV-2: Severe acute respiratory syndrome coronavirus 2
SD: Standard deviation
Q1-Q4: COVID-19-related questionnaires 1 to 4 (Q1 distributed in June 2020; Q2 distributed in December 2020; Q3 distributed in June 2021; Q4 distributed in December 2021)
