# Supplementary material for: Adherence to infection prevention measures and vaccine uptake among pregnant women and new mothers in Sweden during the COVID-19 pandemic
Source: BMC Public Health. 2026 Apr 23;26:1356. doi: 10.1186/s12889-026-27441-x (PMC13107708; doi:10.1186/s12889-026-27441-x)
Supplement: Supplementary file 1 — Additional file 1. The NorthPop timeline [file 12889_2026_27441_MOESM1_ESM.docx]

**Additional file 1. The NorthPop timeline.**

**
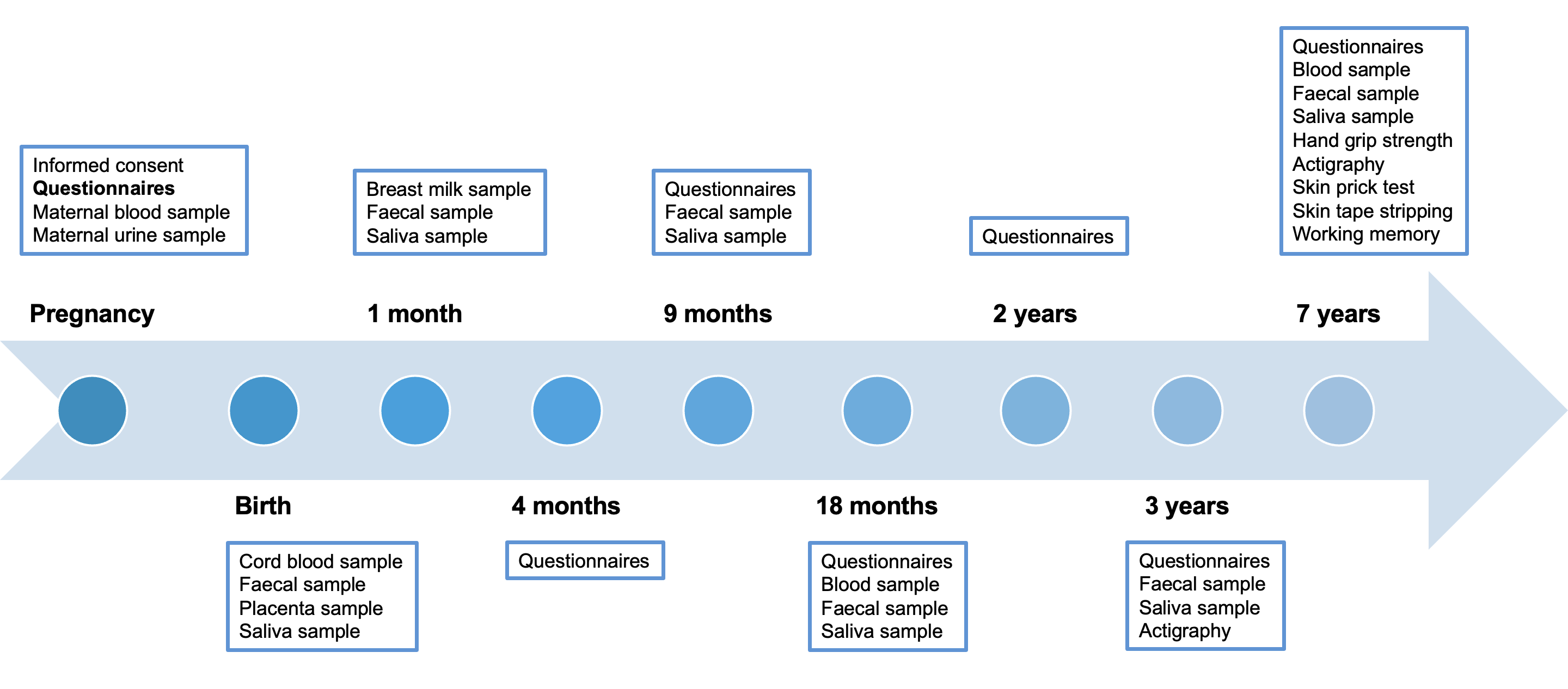
**Additional Fig. 1. This study used responses from the pregnancy questionnaire distributed to the pregnant woman, which is indicated in bold.
