# Supplementary material for: Adherence to infection prevention measures and vaccine uptake among pregnant women and new mothers in Sweden during the COVID-19 pandemic
Source: BMC Public Health. 2026 Apr 23;26:1356. doi: 10.1186/s12889-026-27441-x (PMC13107708; doi:10.1186/s12889-026-27441-x)
Supplement: Supplementary file 2 — Additional file 2. Additional Table 1. Participation analysis in relation to maternal characteristics [file 12889_2026_27441_MOESM2_ESM.docx]

**Additional file 2**.

**Additional Table 1. Participation analysis in relation to maternal characteristics.**

|  | **Original study population** | **Responders** | **Non-responders** |  |
| --- | --- | --- | --- | --- |
|  | N = 3714 | N = 2512 (67.6) | N = 1202 (32.4) |  |
|  |  |  |  | **P-value** |
| Age at questionnaire completion (years), mean (SD)^a^ | | | | |
|  | 32.7 (4.6) | 32.9 (4.5) | 32.4 (4.9) | 0.004 |
| Country of birth | | | | |
| Sweden | 1859 (92.1) | 1647 (88.6) | 212 (11.4) | 0.104 |
| Other country | 159 (7.9) | 134 (84.3) | 25 (15.7) |  |
| Place of delivery | | | | |
| Umeå | 3145 (84.7) | 2087 (66.4) | 1202 (33.6) | < .001 |
| Skellefteå | 471 (12.7) | 362 (76.9) | 109 (23.1) |  |
| Lycksele | 57 (1.5) | 40 (70.2) | 17 (29.8) |  |
| Outside Västerbotten | 41 (1.1) | 23 (56.1) | 18 (43.9) |  |
| Siblings in the household | | | | |
| Yes | 1605 (45.5) | 1094 (68.2) | 511 (31.8) | 0.035 |
| No | 1922 (54.5) | 1373 (71.4) | 549 (28.6) |  |
| Type of housing | | | | |
| House | 1776 (50.4) | 1278 (72.0) | 498 (28.0) | 0.054 |
| Apartment | 1654 (46.9) | 1121 (67.8) | 533 (32.2) |  |
| Farm | 73 (2.1) | 53 (72.6) | 20 (27.4) |  |
| Other | 23 (0.7) | 15 (65.2) | 8 (34.8) |  |
| Highest educational level^b^ | | | | |
| Low | 144 (4.1) | 69 (47.9) | 75 (52.1) | < 0.001 |
| Middle | 931 (26.2) | 591 (63.5) | 340 (36.5) |  |
| High | 2478 (69.7) | 1777 (71.7) | 701 (28.3) |  |
| All participants invited to Q1 are included. Responders were defined as participants who completed the entire questionnaire, and non-responders as those who did not. Data are presented as n (%) unless otherwise indicated. Percentages in the "Original study population" column represent column percentages. Percentages in the "Responders" and "Non-responders" columns represent row percentages (i.e., the proportion of responders/non-responders within each category). P-values are based on Pearson’s chi-square test unless otherwise indicated.  ^a^Independent-samples t-test.  ^b^Low educational level was defined as elementary school or lower, middle educational level as secondary school, and high educational level as university studies. | | | | |
